# Multicolor Emission in Perovskite Nanostructures via Quantum Confinement Engineering for High-Speed Optical Wireless Communication

**DOI:** 10.1021/acsnano.6c05775

**Published:** 2026-05-18

**Authors:** Xin Zhu, Wenqing Niu, Lijie Wang, Xudong Hu, Renqian Zhou, Jian-Xin Wang, Xiaoming Li, Tien Khee Ng, Husam N. Alshareef, Osman M. Bakr, Boon S. Ooi, Omar F. Mohammed

**Affiliations:** † Center of Excellence for Renewable Energy and Storage Technologies, Division of Physical Science and Engineering, 127355 King Abdullah University of Science and Technology, Thuwal 23955-6900, Kingdom of Saudi Arabia; ‡ Photonics Laboratory, Division of Computer, Electrical, and Mathematical Sciences and Engineering, King Abdullah University of Science and Technology, Thuwal 23955-6900, Kingdom of Saudi Arabia; § MIIT Key Laboratory of Advanced Display Materials and Devices, College of Materials Science and Engineering, 12436 Nanjing University of Science and Technology, Nanjing 210094, China

**Keywords:** quantum confinement, perovskite nanostructures, transient absorption spectroscopy, optical wireless communications, multicolor emission

## Abstract

Color converters with broad tunability are critical for achieving large –3-dB bandwidths and high data rates in optical wireless communication (OWC). In this work, quantum confinement engineering is introduced as an effective strategy to design multicolor converters based on lead halide perovskite quantum dots (QDs) for high-speed OWC. Precise control of the size of QDs enables systematic emission tunability across the blue–green region of the visible spectrum, with their efficient photoluminescence and lifetimes of a few nanoseconds providing the wavelength flexibility required for advanced communication protocols. The size-dependent carrier dynamics were examined using transient absorption spectroscopy, confirming their suitability for multichannel operations. Integrating these multicolor converters with narrow emission bandwidth into wavelength-division multiplexing schemes enables data transmission rates of up to 4 Gbps, surpassing most existing color converters employed in OWC systems and demonstrating a practical means toward efficient, scalable, and high-capacity OWC.

## Graphical abstract

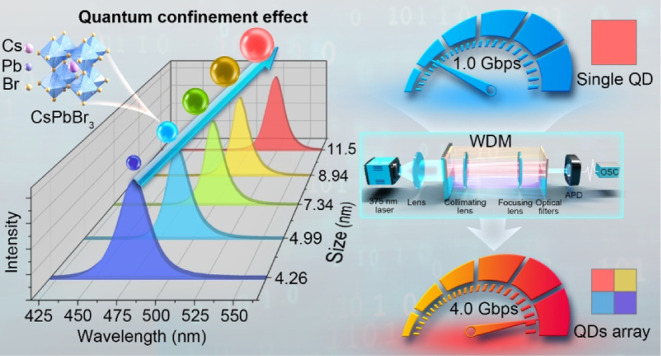

The incredible growth of connected devices, including smartphones, Internet of Things sensors, and autonomous vehicles, has created unprecedented demand for high-speed, secure wireless communication.^1,2^ Conventional radio frequency networks are increasingly limited by spectrum scarcity and network congestion, driving the search for alternative communication technology. Optical wireless communication (OWC) has emerged as a compelling candidate, exploiting the unlicensed and ultrabroad spectral window from ultraviolet to infrared.^3–7^ With its intrinsic features, OWC enables gigabit-level data transmission, offering enhanced security via line-of-sight propagation and the absence of electromagnetic pollution and radio frequency interference.

In practical OWC implementations, color-converting materials are indispensable, as they convert the narrowband emission of laser or light-emitting diodes into broad-spectrum white light with a high color-rendering index, ensuring illumination quality and communication functionality.^8–11^ These materials are crucial for wide-field, large-area photodetection across diverse applications, including underwater links,^12,13^ vehicle-to-vehicle networks,^14,15^ and satellite communication.^16^ Moreover, their photophysical properties critically determine the achievable bandwidth and data rate of OWC, rendering their optimization a principal pursuit in advancing the efficiency, throughput, and scalability of next-generation wireless communication technology.^17^ Conventional phosphors, such as Y_3_Al_5_O_12_:Ce^3+^ (YAG:Ce), offer high brightness and luminous efficiency but have long excited-state lifetimes (on the microsecond scale), which limit their application in high-speed optical communication.^18,19^ In addition, most other phosphor-based color converters exhibit similar limitations, such as broad emission peaks,^20^ which can compromise their performance in other OWC applications, such as multichannel optical communication. Furthermore, organic color converters often involve complex synthetic procedures,^21^ posing additional challenges for large-scale, cost-effective fabrication. Therefore, identifying an appropriate strategy to overcome these limitations is of critical importance for the development of high-performance and scalable OWC systems.

Quantum confinement engineering offers a powerful strategy to address this challenge by precisely controlling the size of perovskite nanocrystals without complex synthetic procedures, enabling the systematic tuning of their emission wavelengths across the visible spectrum and PL lifetime.^22–26^ This strategy provides a flexible way to tune the emission spectrum without introducing additional materials or suffering from the phase separation commonly observed in mixed-halide systems. In contrast, conventional halide-composition engineering routes lack the ability to tune the photoluminescence (PL) lifetime, a key parameter for high-speed OWC. The size-dependent tunability facilitates the development of multicolor emission converters, which are advantageous for implementing wavelength-division multiplexing (WDM) in OWCs.^27–29^ Their intrinsically narrow emission bandwidths minimize spectral overlap and crosstalk between adjacent channels, ensuring superior channel isolation. When combined with WDM, multicolor converters allow dense channel packing, enabling multiple independent data streams to transmit simultaneously over distinct optical channels, improving spectral efficiency and significantly boosting data throughput.

To implement this strategy, metal halide perovskites were chosen as a model system due to their remarkable multifunctionality in various applications, including photovoltaics, light-emitting devices, and energy storage, owing to their solution processability, low-cost fabrication, and efficient luminescence.^30–33^ Beyond this fundamental advantage, their superior photophysical properties (e.g., high PL quantum yields (PLQYs), nanosecond-scale lifetimes, and narrow emission bandwidths) further underscore their potential for integration into next-generation communication architectures.^34–38^ Most importantly, metal halide perovskites allow well-controlled size tunability and desirable emission behavior through quantum confinement engineering, providing an ideal platform for high-performance multicolor converters. Although CsPbX_3_-based OWC systems have been explored, primarily through halide composition engineering of single-color NCs. These methods often involve complex synthesis and delivered limited performance with reported data rates of approximately100 Mbps and bandwidths of around 15 MHz.^37^ Moreover, they have not deliberately optimized the photophysical properties of the nanocrystals to enhance OWC efficiency.

Thus, this work introduces quantum confinement engineering as an effective strategy to develop multicolor converters based on lead halide perovskites for high-speed OWC applications. By precisely controlling the size of CsPbBr_3_ perovskite-based quantum dots (QDs), their emissions can be tuned across the visible spectrum, exhibiting efficient PL and lifetimes of a few nanoseconds, enabling wavelength flexibility that is crucial for advanced communication schemes. The dynamics of QDs of varying sizes were studied using transient absorption (TA) spectroscopy. The resulting multicolor converters with narrow emission bandwidths demonstrate the potential to achieve data transmission rates of up to 4 Gbps with the integration of WDM (Figure 1), significantly improving bandwidth utilization. This approach offers a practical pathway toward efficient, scalable, and high-capacity OWC systems.

**Figure 1:**
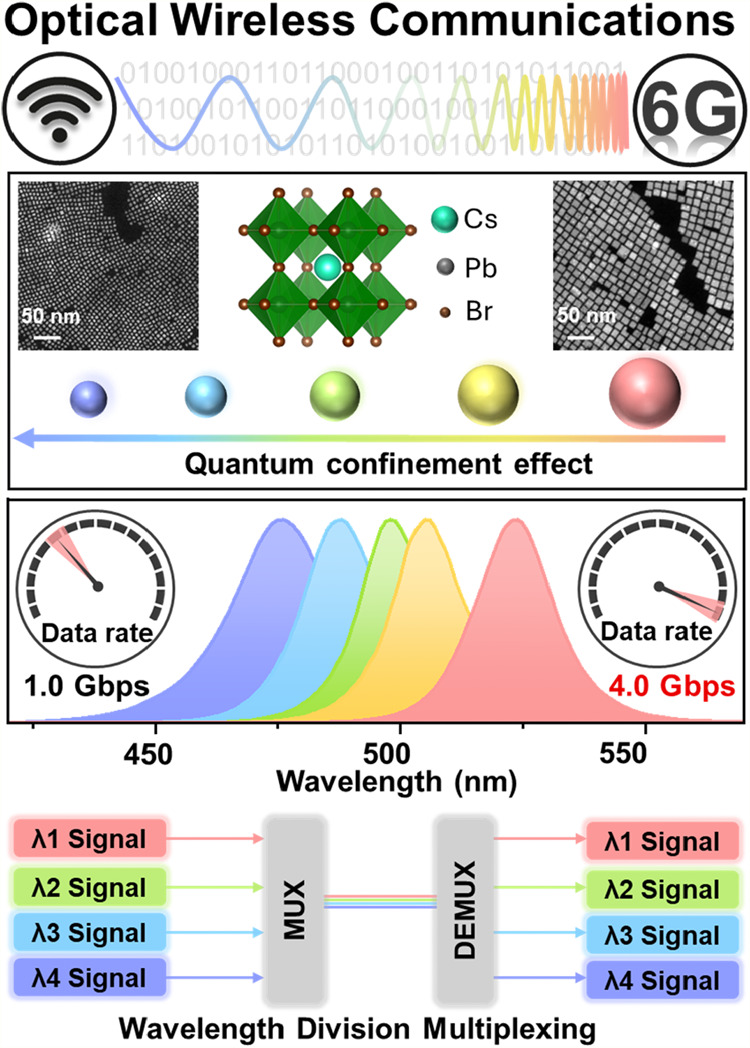
Schematic of the advantages of CsPbBr_3_ quantum dots with a quantum confinement strategy for high-performance optical wireless communication applications.

## Results and Discussion

The cube-shaped CsPbBr_3_ perovskite QDs were synthesized following the procedures described in the Methods section. Their crystalline phase and phase purity were confirmed using X-ray powder diffraction, with the observed diffraction peaks in agreement with the simulated cubic CsPbBr_3_ pattern (Figure 2a). Transmission electron microscopy revealed that the edge length (*L*) of the perovskite QDs could be precisely tuned from 4.2 to 11.7 nm (Figures 2b,c and S1). Notably, this tunable range encompasses the Bohr exciton diameter of about 7 nm for CsPbBr_3_, enabling a systematic investigation of the quantum confinement effects.^36^

**Figure 2:**
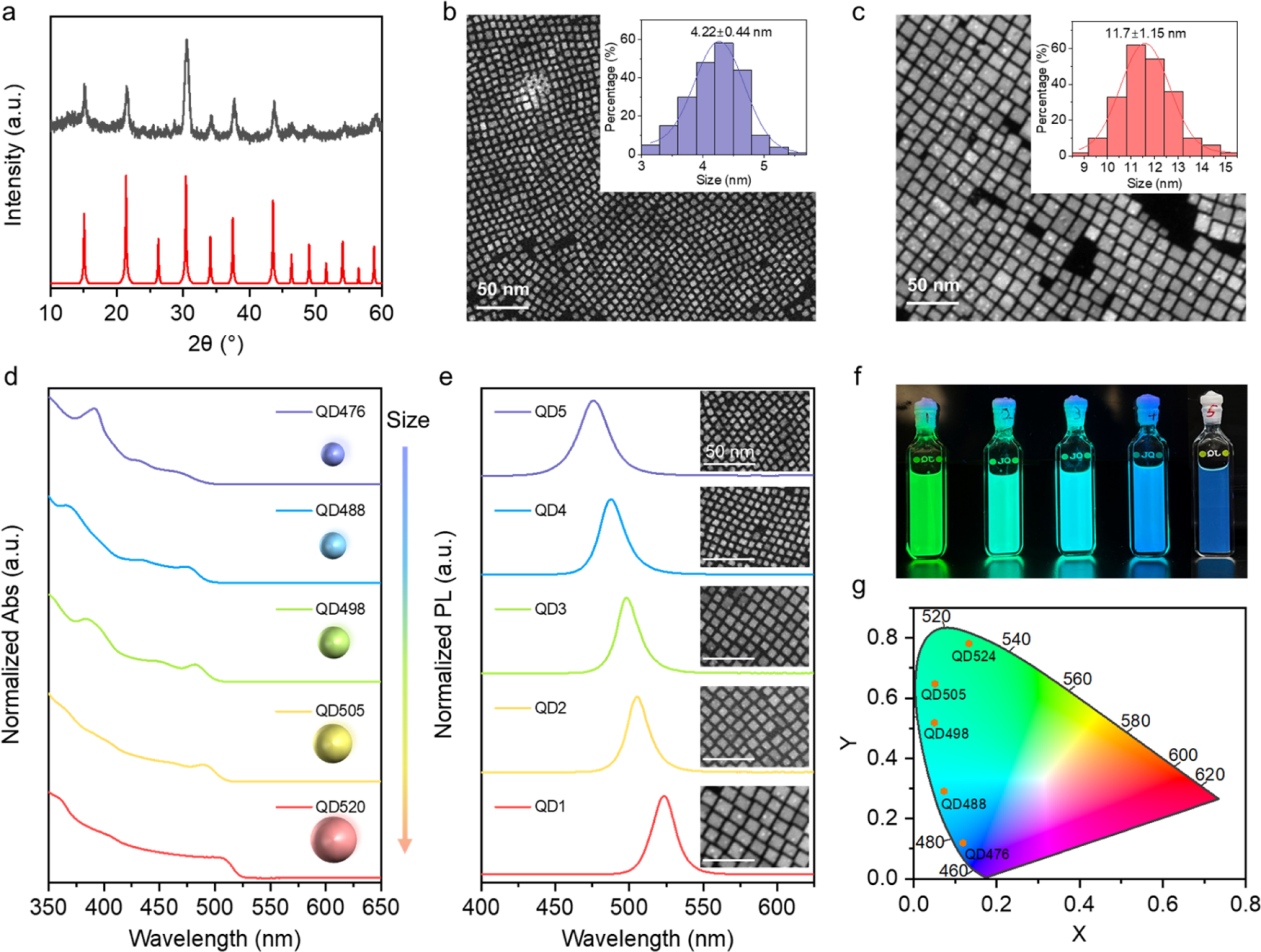
(a) Comparison of X-ray powder diffraction patterns of CsPbBr_3_ QD (black line) with the simulated patterns (red line). Transmission electron microscopy and size distribution histogram of CsPbBr_3_ QD5 (b) and QD1 (c) (215 particles analyzed per sample). Absorption (d) and emission (e) spectra with varying QD sizes. (f) CsPbBr_3_ QD solution in hexane. (g) The CIE 1931 color coordinates of varying QD Size.

As expected, the absorption spectra presented a pronounced blue shift in the lowest energy excitonic transition from 507 to 464 nm as *L* decreased from 11.7 to 4.2 nm (Figure 2d), consistent with the strong quantum confinement regime. Upon excitation at 375 nm, the QDs exhibited PL with emission peaks ranging from 524 to 476 nm (Figure 2e). The CsPbBr_3_ QDs in hexane solution displayed a visible color change from green to blue (Figure 2f), with PLQYs of 76–46% (Table S1), indicating high emission efficiency across the studied size range. In addition, the quantum confinement effect induced a blue shift in absorption and emission, resulting in a shortened PL lifetime as the size of the QDs decreased (Figure S2). The PL color purity of the QDs was further evaluated using the CIE 1931 chromaticity diagram. As in Figure 2g, the emission peaks corresponding to various QD sizes are well separated across the visible region, and their CIE coordinates lie near the edge of the color space, which is indicative of narrow emission bandwidth and high color saturation. Such distinct color coordinates highlight the potential of these QDs for applications in multicolor display applications. Moreover, QD1 and QD5 retained over 95% of their initial PL intensity under continuous UV laser irradiation (Figure S3a and b). Thermal stability tests showed that PL intensity remained above 94% for QD1 and 88% for QD5 (Figure S3c and d), confirming their strong resistance to thermal degradation. These results demonstrate promising light and thermal stability of these perovskite QDs.

To clarify how quantum confinement modulates the photophysical behavior of CsPbBr_3_ QDs, TA spectroscopy was performed on perovskite QDs of various sizes to probe their excited-state dynamics. Figures 3a and S4 present the experimental TA map plotted as Δ*A* versus the wavelength and delay time. Figure S5 illustrates the corresponding TA spectral traces by time delay. A pronounced bleach feature centered at 500 to 510 nm appears immediately after excitation and persists from 0.2 ps to 3 ns, which is consistent with excitonic bleaching^39^ and is accompanied by weaker signals on the short- and long-wavelength sides. Figure 3b displays the results of the global lifetime density analysis, which condenses the data set into a lifetime-resolved map,^40,41^ described as follows:

$$S\left(t,\lambda_{exc},\lambda_{pro}\right)=\sum_{j=1}^{n}A_{j}\left(\tau ,\lambda_{exc},\lambda_{pro}\right)\exp\left(-t/\tau_{j}\right)⊗IRF\left(t\right)$$

where τ represents the global lifetime, *A*_*j*_ denotes the amplitude, and IRF­(*t*) indicates the instrument response function (∼200 fs in this setup).^42^ Here, “⊗” denotes convolution, and the instrument response function (IRF) was obtained from pump–probe cross-correlation measurements using the instantaneous cross-phase modulation signal. This multidimensional analysis captures the intrinsic data complexity, enabling the deconvolution of nonexponential kinetics and spectrally congested features, resolving the decay components and uncovering energy- or charge-transfer pathways that are not accessible via conventional time-trace analyses. The close match between the fitted and experimental maps (residuals in Figure S6) confirms that the kinetic model accurately reproduces the temporal-spectral behavior.

**Figure 3:**
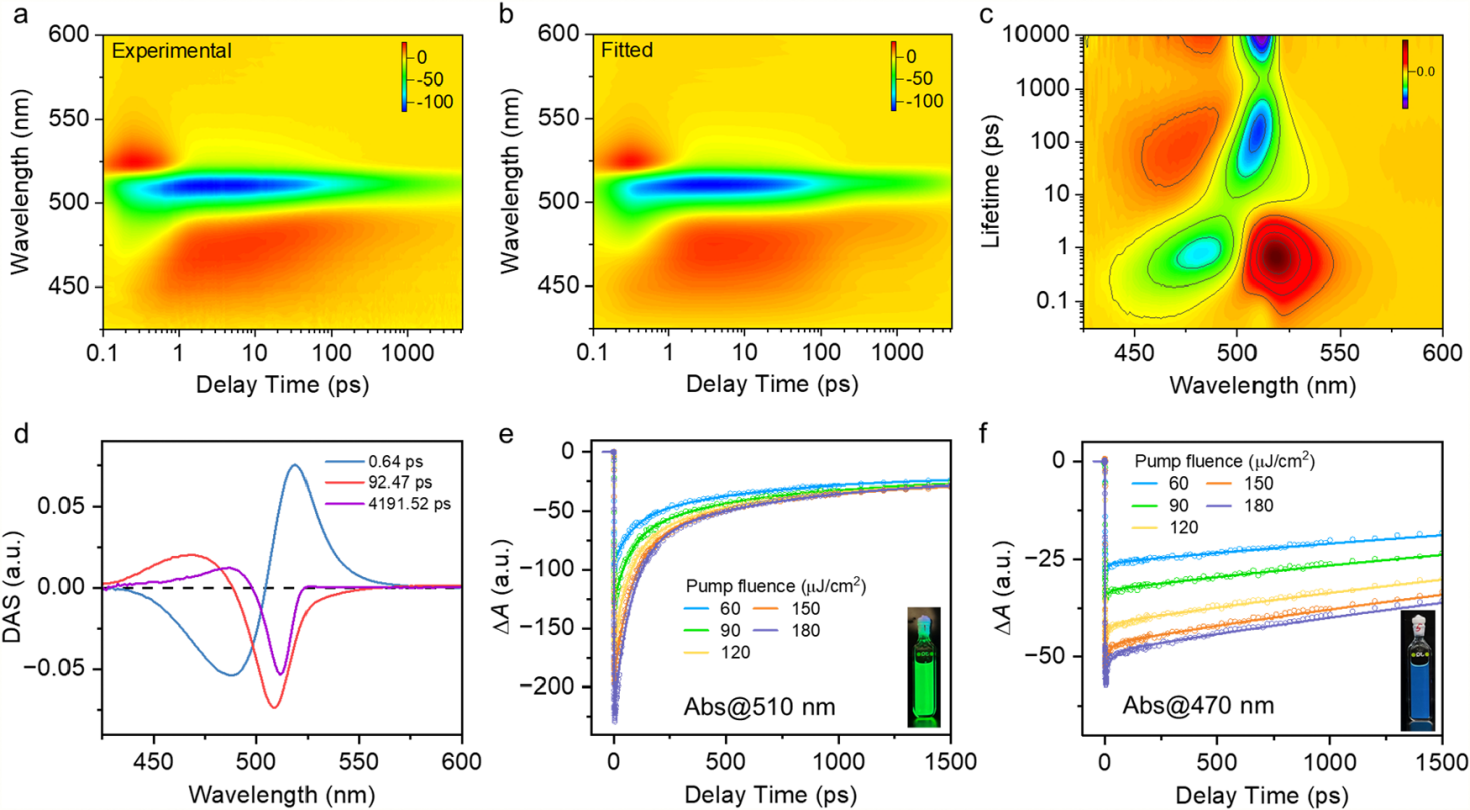
(a) Experimental TA time-wavelength map of CsPbBr_3_ QD1 probed in the visible spectral region. (b) Fitted TA map obtained using a global lifetime analysis. (c) Lifetime density distribution map in the lifetime range of 0.03 to 10,000 ps. (d) Generated decay-associated spectra (DAS) at corresponding lifetimes, resulting from the global lifetime analysis. Pump fluence-dependent TA kinetics of CsPbBr_3_ QDs at selected probe wavelengths. (e) Largest QD (QD1) probed at 510 nm. (f) Smallest QD (QD5) probed at 470 nm.

The corresponding lifetime density distribution (Figure 3c) has three distinct time domains, and it assumes 50 lifetime components ranging from 0.03 to 10,000 ps, with high-intensity regions indicating the most probable lifetimes at each probe wavelength. This decomposition reveals that dynamic processes govern relaxation, with an ultrafast component (<1 ps), intermediate decays on the time scale of tens to hundreds of ps, and a long-lived channel extending beyond the measurement window, each with a characteristic spectral evolution and shift. Decay-associated spectra (Figure 3d) for representative lifetimes of 0.64 ps, 92.5 ps, and 4.19 ns illustrate these dynamics. The earliest component displays a sharp derivative-like profile around the bleach region, which is characteristic of bandgap renormalization immediately after photoexcitation.^41^ In contrast, the slower components are centered in the band- edge bleach region but show distinct spectral distributions. The intermediate component appears somewhat broader and slightly blue-shifted relative to the long-lived component, whereas the long-lived component is more localized around the bleach minimum. These components are assigned primarily to multiexcitonic relaxation and to residual single-exciton/trap-associated decay, respectively.

Fluence-dependent TA kinetics were examined across the full QD size series at the bleach maximum of each sample (Figures 3e,f and S7–S8). For all QDs, increasing pump fluence enhances the bleach amplitude and modifies the subsequent recovery, indicating that higher excitation density changes the relative contribution of the transient relaxation channels. However, the way the kinetics respond to fluence evolves systematically with QD size. The largest QD (QD1) exhibits a strongly fluence-dependent, multitime constants bleach recovery, in which the early time recovery contribution becomes markedly more pronounced at higher excitation density. By contrast, as the QD size decreases, the normalized bleach kinetics become progressively less sensitive to fluence and evolve toward a more persistent slow-recovery profile. In the smallest QD regime, the traces at different fluences become much more similar in shape, indicating that the long-time bleach recovery is increasingly governed by a common slow channel.

A direct comparison across the size series at the same pump fluence further reveals a clear kinetic crossover from a fast early recovery dominated response in large QDs to a more persistent slow-bleach regime in small QDs. This behavior is consistent with an increased contribution from carrier reheating and lattice-coupled relaxation processes in the strongly confined QDs, potentially including Auger-reheating related effects. These results show that decreasing QD size does not simply accelerate the entire bleach recovery uniformly; rather, it changes how excitation density is manifested in the band-edge transient response by redistributing the relative weight of early time and long-lived recovery contributions.^43^

The CsPbBr_3_ QDs with size-dependent emission colors exhibit excellent photoluminescent efficiency and PL lifetimes of a few nanoseconds, making them promising candidates for OWC applications. To demonstrate this potential experimentally, the performance of perovskite QDs was evaluated in the OWC channel in Figure S9. The system comprises a digital signal source, an arbitrary waveform generator, an electrical amplifier, and a bias-tee that combines the direct current (DC) bias with the alternating current (AC) modulation signal. The modulated electrical signal drives a 375 nm laser diode to generate optical signals, which are focused on the perovskite QD solution samples sealed in the cuvette. The emitted visible light is collected and directed onto an avalanche photodetector (APD) via an optical filter. The detected signals are recorded using an oscilloscope for off-line processing and bit-error-rate (BER) analysis.

The −3-dB bandwidth for the perovskite QDs was extracted from the frequency response results by applying sinusoidal AC signals (Figure S10), revealing bandwidths of about 35 MHz for all samples (Figure 4a). Among these samples, QD1 demonstrated a slower roll-off beyond the −3-dB. Although the modulation of bandwidth is primarily governed by the PL lifetime, it can also be influenced by other photophysical factors. In this size series, the shorter PL lifetimes of the smaller-emission QDs are consistent with their faster emissive response, while the concurrently reduced PLQY indicates a stronger nonradiative contribution. These competing effects likely contribute to the relatively similar measured bandwidths across the samples.^44^ Figure 4b summarizes the corresponding net data rates and BER results. All measured BER values remain below the hard-decision forward-error-correction (FEC) threshold of 3.8 × 10^–3^. The net data rates were obtained under identical conditions of incident ultraviolet light power and signal peak-to-peak voltage (*V*_pp_). The achievable net data rate is not determined solely by the −3-dB bandwidth; it is also influenced by the luminescence efficiency of the QDs, affecting the emitted optical power.

**Figure 4:**
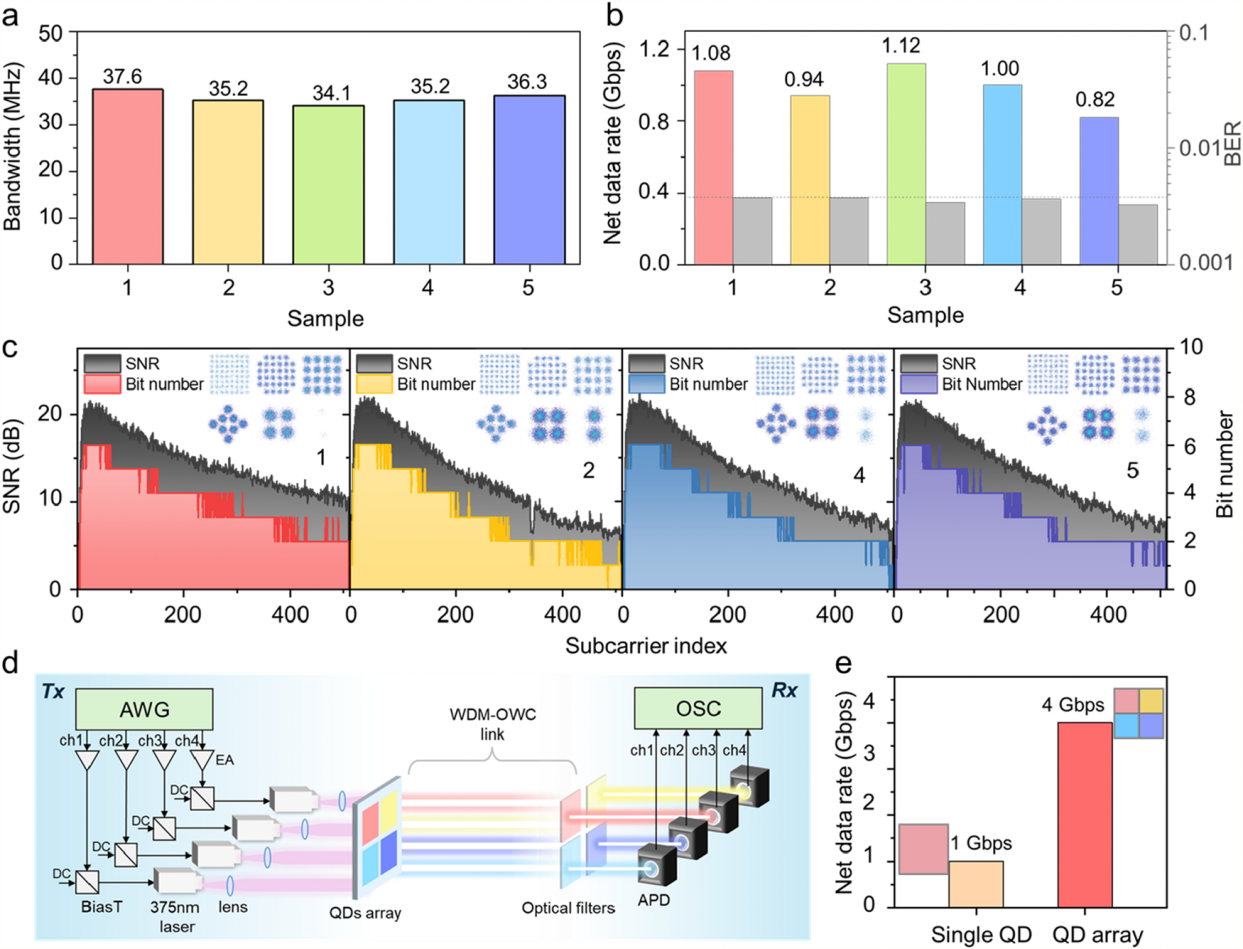
(a) The −3-dB bandwidth by varying emission QDs. (b) Net data rate by varying emission QDs. (c) Signal-to-noise ratio (SNR) estimation and corresponding constellation diagrams of received signals (insets) by varying emission QDs. (d) Schematic of the wavelength-division multiplexing (WDM) system. AWG: arbitrary waveform generator; EA: Electrical amplifier; APD: avalanche photodetector; OSC: oscilloscope; Tx: transmitter; Rx: receiver. (e) Net data rate comparison of the single QD and QD array.

Furthermore, this approach applies bit-and-power-loading, discrete-multitone modulation to employ the available bandwidth fully. The signal-to-noise ratio (SNR) is estimated before implementing the bit-and-power allocation, which is based on the error vector magnitude (EVM) of the quadrature phase-shift keying signal. Figures 4c and S11 depict the estimated SNR of each subcarrier and the bit number allocation results, and Figure S12 presents the allocated power ratio results by sample. Notably, the Levin–Campello algorithm was adopted for the joint optimization of the bit number and power allocation^45^ which is expected to achieve higher spectral efficiency than the conventional water-filling strategy. The corresponding constellation diagrams of the received signals are displayed as insets. The maximum bit number that can be achieved is 6 bits per symbol, corresponding to a 64-quadrature amplitude modulation (QAM). In addition, a geometrically shaped 8-QAM with a diamond distribution was employed to replace the conventional in-phase/quadrature-unbalanced 8-QAM. Figure S13 displays the electrical spectra of the received signals from different emission perovskite QDs. The sampling rates for QD1–QD4 were 1.2 GSa/s, whereas that for QD5 was 1.0 GSa/s. The electrical spectra were obtained by performing a fast Fourier transform (FFT) on the time-domain signals. The corresponding bandwidths were determined to be 300 and 250 MHz, respectively. Notably, QD1 exhibits a more gradual spectral roll-off, as shown in Figure S13a, which is consistent with its frequency response behavior presented in Figure S10.

The multicolor emission and narrow spectral bandwidths of CsPbBr_3_ QDs offer significant advantages for WDM, as distinct emission bands enable spectral separation between channels, improving spectral efficiency in high-capacity optical communication. To demonstrate this potential, the QD1, QD2, QD4, and QD5 samples were integrated into a 2 × 2 array for WDM operation. Figure 4d presents a conceptual schematic of the WDM-based OWC system. In this configuration, multiple independent electrical inputs are amplified, combined with the DC bias via bias-tees, and employed to drive the individual laser diodes. Each laser diode excites a distinct region of the integrated QD array containing different emissive materials. On the transmitting side, a specially designed lens array is employed to achieve efficient optical collimation. After free-space propagation, the transmitted signals are collected using separate APDs. To minimize crosstalk between adjacent wavelength channels, narrow-bandpass optical filters are placed in front of each APD. The detected signals are demodulated individually via off-line digital signal processing. Figure S14 presents the spectral allocation and crosstalk analysis for QD-based WDM channels with commercially available bandpass filters. Although the emission line widths of QDs are broader than those of laser sources, their relatively well-defined spectral profiles allow coarse WDM operation when combined with appropriate optical filtering and signal processing. Therefore, the WDM-OWC system is capable of supporting a maximum achievable aggregate data rate of up to 4 Gbps (Figure 4e), compared with that of a single QD channel, and outperforms those of most QD-based materials.^46–48^ These findings demonstrate the significant potential of multicolor emission materials for high-speed, multiuser OWC applications.

## Conclusions

In conclusion, this work demonstrates that quantum confinement engineering is an effective approach for developing multicolor converters based on lead halide perovskites for high-speed OWC applications. By controlling the size of QDs through quantum confinement, their emission wavelengths can be precisely tuned across the visible spectrum, producing strong PL and short lifetimes that are suitable for advanced communication schemes. The TA spectroscopy results revealed the excited-state dynamics of QDs by varying sizes, providing insight into their photophysical processes. The QD-based multicolor converters with narrow emission bandwidth enabled data transmission rates of up to 4 Gbps via WDM integration, highlighting their potential to enhance bandwidth utilization and offering a promising route toward practical, efficient, and scalable high-capacity OWC systems.

## Methods

All chemicals were purchased from commercial suppliers (Sigma-Aldrich) and used without further purification. The UV–vis absorption measurements were performed by using a Cary 5000 UV–vis–NIR spectrometer. PerkinElmer LS45 Photoluminescence spectrometer equipped with a 450 W xenon lamp was used for steady-state photoluminescence studies. Powder X-ray diffraction (PXRD) patterns were collected by a Bruker D8 ADVANCE diffractometer for Cu Kα radiation (λ = 1.5406 Å). Transmission electron microscopy (TEM) analysis was carried out with Titan (FEI Company) operating at a beam energy of 300 keV and equipped with a Tridiem postcolumn energy filter (Gatan, INC.). Photoluminescence quantum yield (PLQY) measurements were also carried out on QD solutions at room temperature, using an Edinburgh FS5 fluorescence spectrometer equipped with an SC-30 integrating sphere.

### Synthesis of Perovskite Quantum Dots

#### Precursor Preparation

Specifically, 0.25 g of Cs_2_CO_3_, 0.9 mL oleic acid (OA), and 8.87 mL 1-octadecene (ODE) were loaded into a 50 mL three-neck flask and vacuum-dried at 120 °C for 1 h using a Schlenk line. The mixture was then heated to 150 °C under an Ar atmosphere until all Cs_2_CO_3_ powders were completely dissolved. To prevent precipitation of Cs-oleate from ODE, the precursor solution was maintained at 100 °C before subsequent use.

#### QDs Preparation

In a separate 100 mL three-neck flask, the PbBr_2_ precursor solution was prepared by dissolving 75 mg of PbBr_2_ together with varying amounts of ZnBr_2_ (0–600 mg) in a mixture of ODE (5 mL), OA (3.5 mL), and oleylamine (OAm, 3.5 mL). The solution was heated at 120 °C for 1 h under an Ar atmosphere. Thereafter, 0.4 mL of the Cs-oleate precursor was swiftly injected to initiate nucleation and growth. The reaction was quenched after 10–144 s (depending on the reaction temperature, 180–100 °C. e.g., 10 s/0 mg for green QDs, 144 s/480 mg for blue QDs) by cooling the flask in an ice bath. Upon cooling to 60 °C, the crude suspension was washed by 3:1 volume ethyl acetate at 3500 rpm for 5 min to remove unreacted solvent and ligands, and the perovskite QDs were dispersed in the hexane then collected.

### Time-Resolved Transient Absorption Spectroscopy

TA measurements were conducted using a Helios pump–probe setup (Ultrafast Systems), enabling broadband visible probing upon tunable visible excitation of the samples. A 1 kHz, 800 nm, ∼150 fs, 7 mJ laser pulse was split into two parts for the pump and probe. The pump was generated using a nonlinear optical parametric amplifier (SpectraPhysics), delivering pulses from ∼350 to ∼700 nm, while the probe was created by focusing the remaining 800 nm beam into a 2 mm CaF_2_ crystal. The white-light probe beam was further split into signal and reference paths, with the reference beam used to improve the signal-to-noise ratio. The pump and probe beams were spatially overlapped on the sample, and the pump–probe delay was controlled by a motorized optical delay line in the Helios system. The transmitted probe signal was collected and focused into a fiber-coupled detector. In the experiment, the typical pump and probe spot sizes are 120 and 60 μm in diameter, respectively. The pump fluences were identical for all samples, ranging from 60 μJ/cm^2^ at the lowest excitation level up to 180 μJ/cm^2^ for the highest fluence. All samples were measured in 1 mm path length quartz cuvettes with comparable concentrations.

### OWC Measurement

The transmitting signals were generated offline using MATLAB, and the digital signals were converted into analog waveforms by the arbitrary waveform generator (AWG, Tektronix AWG70002A). Then an electrical amplifier (EA, Mini-Circuits, ZHL-2–8-S+) was utilized to amplify the signal, followed by a bias-Tee, which combined the alternating current (AC) signal with the direct current (DC) bias. A 375 nm laser diode (LD) converted the electrical signals into optical signals. The LD was mounted on a laser control system (Thorlabs, LDM56 and ITC4005) for temperature and current stabilization. A focusing lens directs the UV laser beamed onto the perovskite QD solution sample. Afterward, the excited visible light was collimated by a collimating lens.

At the receiving side, a lens was used to focus the beam onto an avalanche photodetector (APD, Thorlabs, APD430A2/M). An optical filter was placed before the APD to remove the 375 nm UV light. The detected analog signals were then captured by an oscilloscope (OSC, Tektronix DPO72004C), which converted them into digital signals for offline processing. Finally, the bit error rate (BER) was calculated through digital signal processing. The frequency response was measured using a network analyzer (Agilent, E5061B).

## Supplementary Material



## Notes

Details are provided on figures depicting transmission electron microscopy images, photophysical performance, stability measurements, TA data, and optical wireless communication performance (PDF)
