# Supplementary material for: Multicolor Emission in Perovskite Nanostructures via Quantum Confinement Engineering for High-Speed Optical Wireless Communication
Source: ACS Nano. 2026 May 18;20(21):15744–51. doi: 10.1021/acsnano.6c05775 (PMC13235639; doi:10.1021/acsnano.6c05775)
Supplement: Supplementary file 1 [file nn6c05775_si_001.pdf]

Supporting Information

Multicolor Emission in Perovskite Nanostructures *via* Quantum Confinement Engineering for High-Speed Optical Wireless Communication

Xin Zhu[†⊥], Wenqing Niu[‡⊥], Lijie Wang[†⊥], Xudong Hu[†§⊥], Renqian Zhou[†], Jian-Xin Wang[†], Xiaoming Li[§], Tien Khee Ng[‡], Husam N. Alshareef[†], Osman M. Bakr[†], Boon S. Ooi[‡*], Omar F. Mohammed[†*]

†Center of Excellence for Renewable Energy and Storage Technologies, Division of Physical Science and Engineering, King Abdullah University of Science and Technology, Thuwal 23955-6900, Kingdom of Saudi Arabia

‡Photonics Laboratory, Division of Computer, Electrical, and Mathematical Sciences and Engineering, King Abdullah University of Science and Technology, Thuwal 23955-6900, Kingdom of Saudi Arabia

§MIIT Key Laboratory of Advanced Display Materials and Devices, College of Materials Science and Engineering, Nanjing University of Science and Technology, Nanjing 210094, China

*Corresponding Authors: Boon S. Ooi, Email: boon.ooi@kaust.edu.sa; Omar F. Mohammed, Email: omar.abdelsaboor@kaust.edu.sa

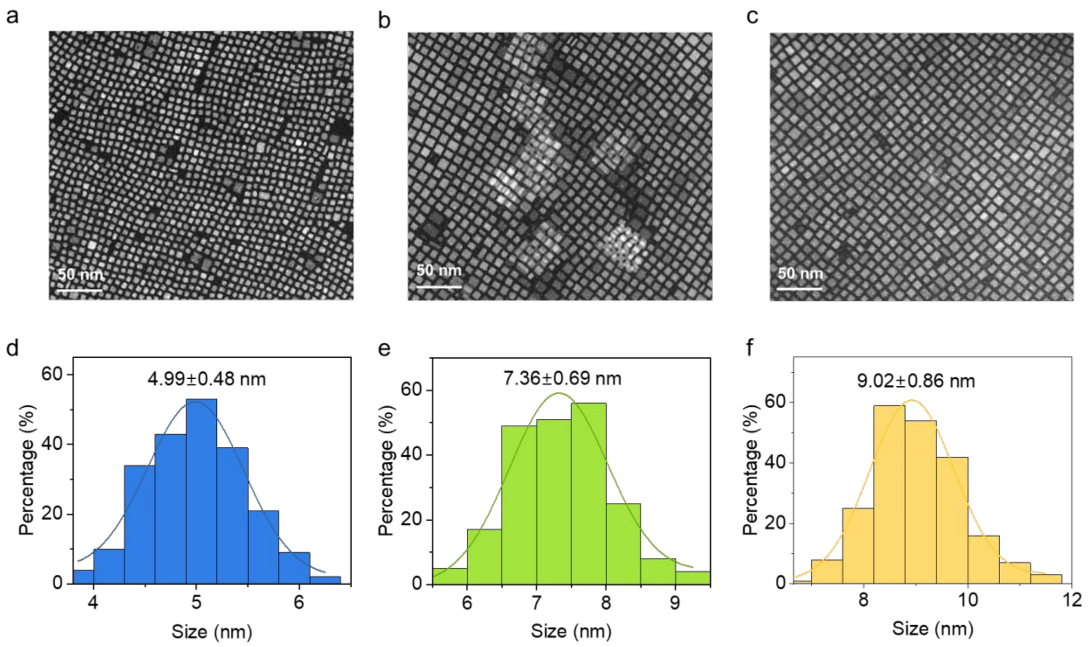

Figure S1. TEM and size distribution histogram of $CsPbBr_3$ QDs with varying, with 215 particles analyzed for each sample.

Table S1. Fluorescence quantum yield (PLQY) of $CsPbBr_3$ QDs with varying sizes.

Sample	QD1	QD2	QD3	QD4	QD5
PLQY (%)	76	66	51	42	47

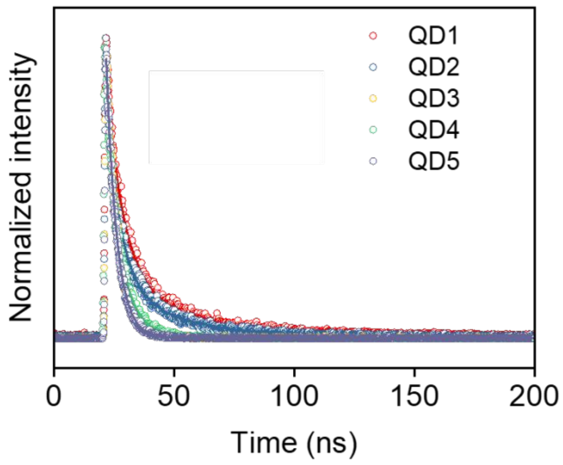

Figure S2. The time-resolved emission decays of $CsPbBr_3$ QDs with varying sizes.

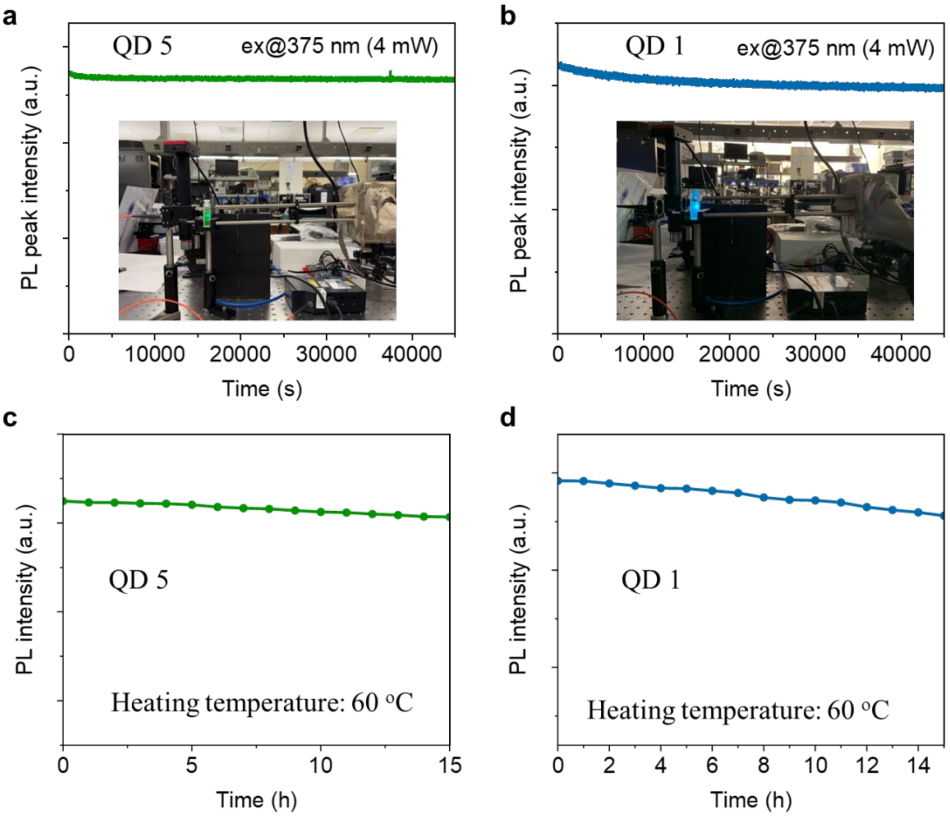

Figure S3. Stability measurements of $CsPbBr_3$ QDs. Laser radiation stability results of (a) QD5 and (b) QD1. The inset pictures demonstrate the experimental set-up. Thermal stability results of (c) QD5 and (d) QD1. Heating temperature is 60 ^{o}C.

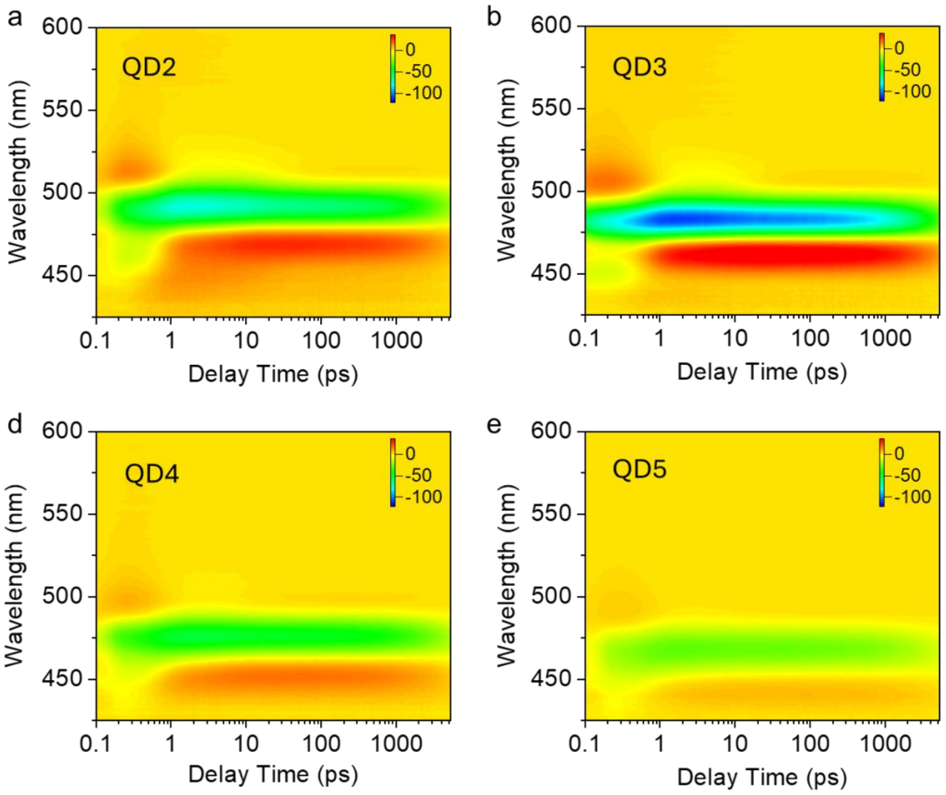

Figure S4. Experimental TA time-wavelength map of $CsPbBr_3$ QDs with varying sizes probed in the visible spectral region from 425-600 nm.

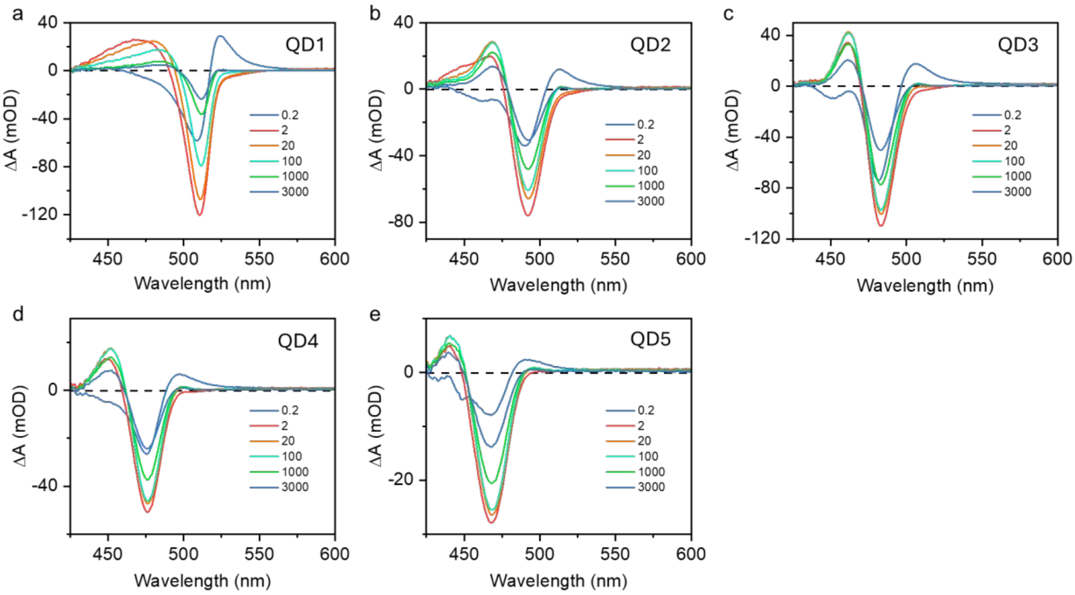

Figure S5. TA spectral traces of $CsPbBr_3$ QDs with varying sizes at 0.2, 2, 20, 100, 1000, and 3000 ps, respectively.

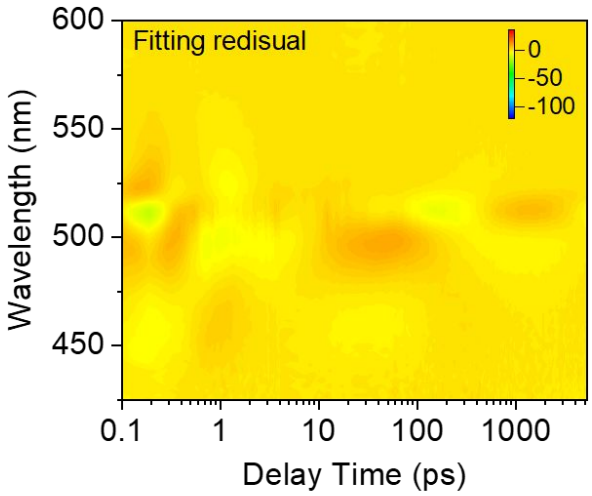

Figure S6. The residuals map with the global lifetime fitting of QD1.

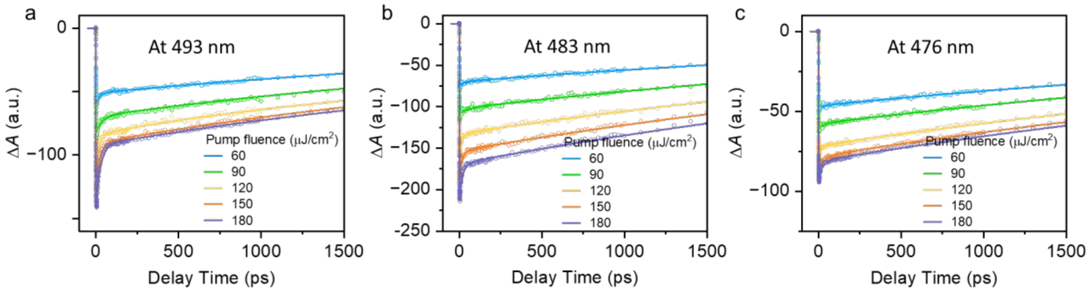

Figure S7. Pump fluence–dependent TA kinetics of $CsPbBr_3$ QDs at selected probe wavelengths. QDs probed at 493 nm (a), 483 nm (b), and 476 nm (c), respectively.

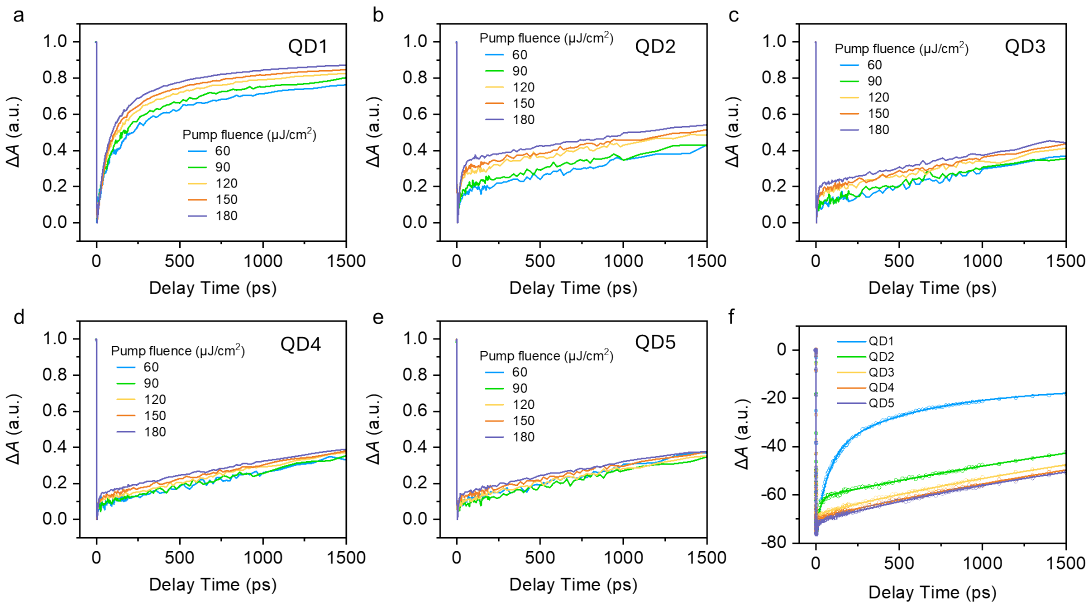

Figure S8. Pump fluence-dependent normalized TA kinetics of the CsPbBr3 QD size series at the bleach maximum of each sample. (a-e) Normalized bleach recovery traces of QD1–QD5, respectively, at different pump fluences. (f) comparison of the bleach kinetics of all five QDs at the same pump fluence (60 μJ/cm^2), showing the systematic evolution of the recovery profile with decreasing QD size.

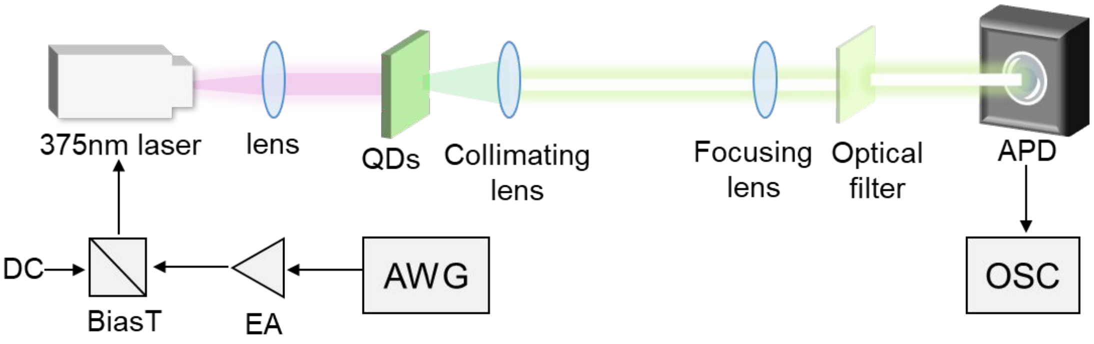

Figure S9. Schematic representation of the OWC channel.

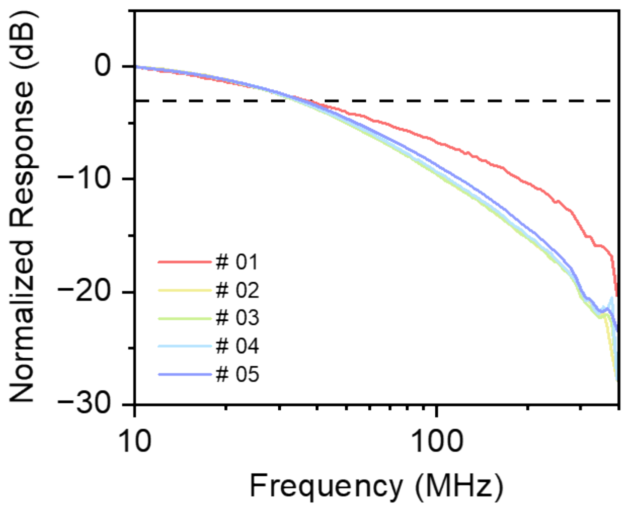

Figure S10. Normalized frequency responses of varying emission QDs.

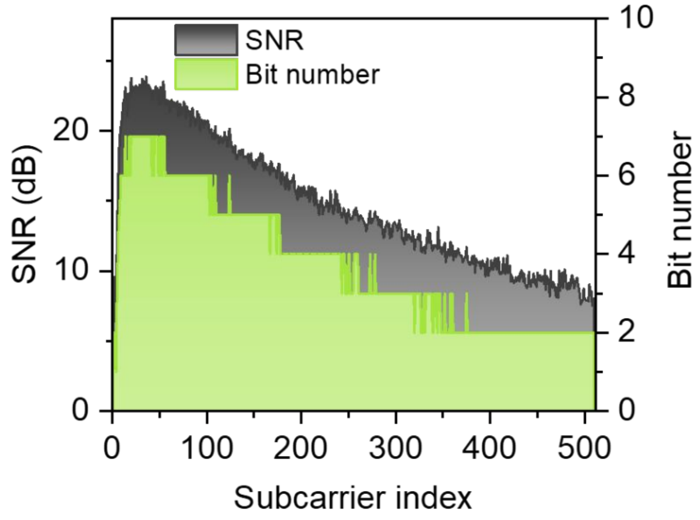

Figure S11. The SNR and the corresponding allocated bit number for QD3.

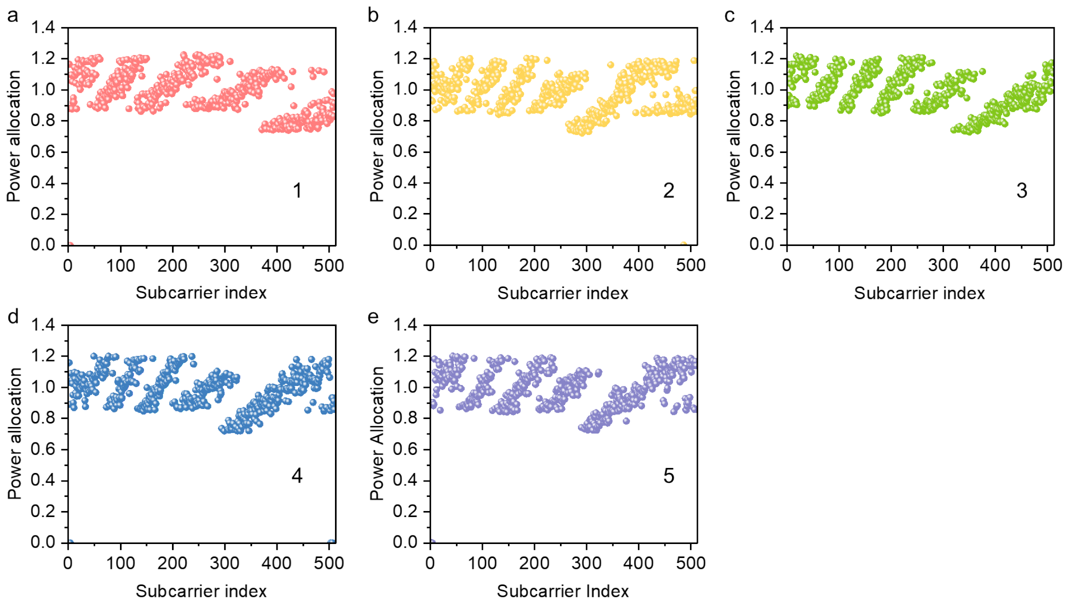

Figure S12. Power allocation of varying emission QDs.

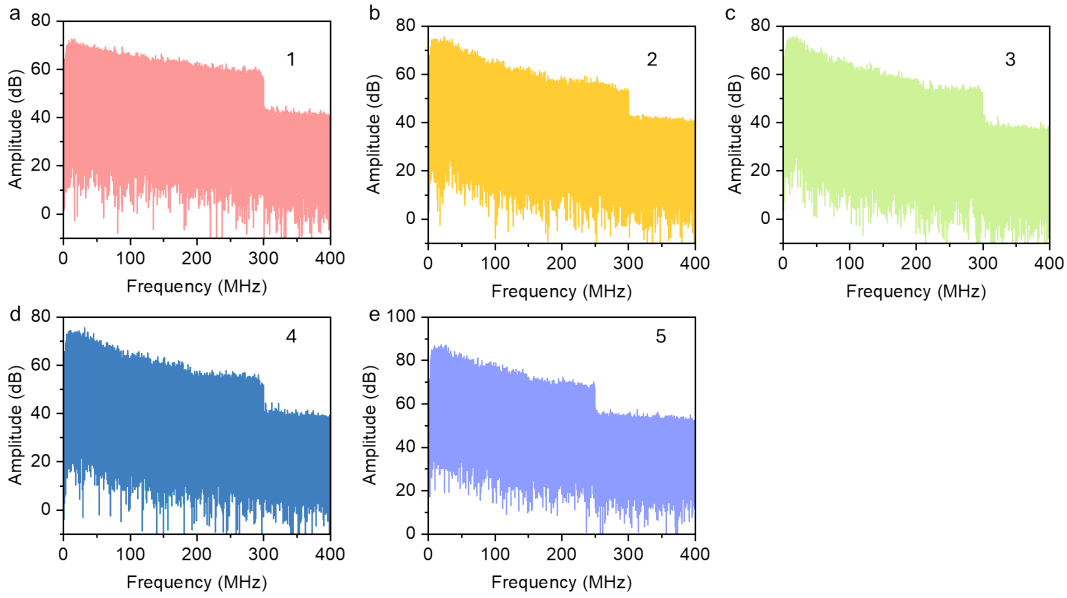

Figure S13. Electrical spectrum of the received signal.

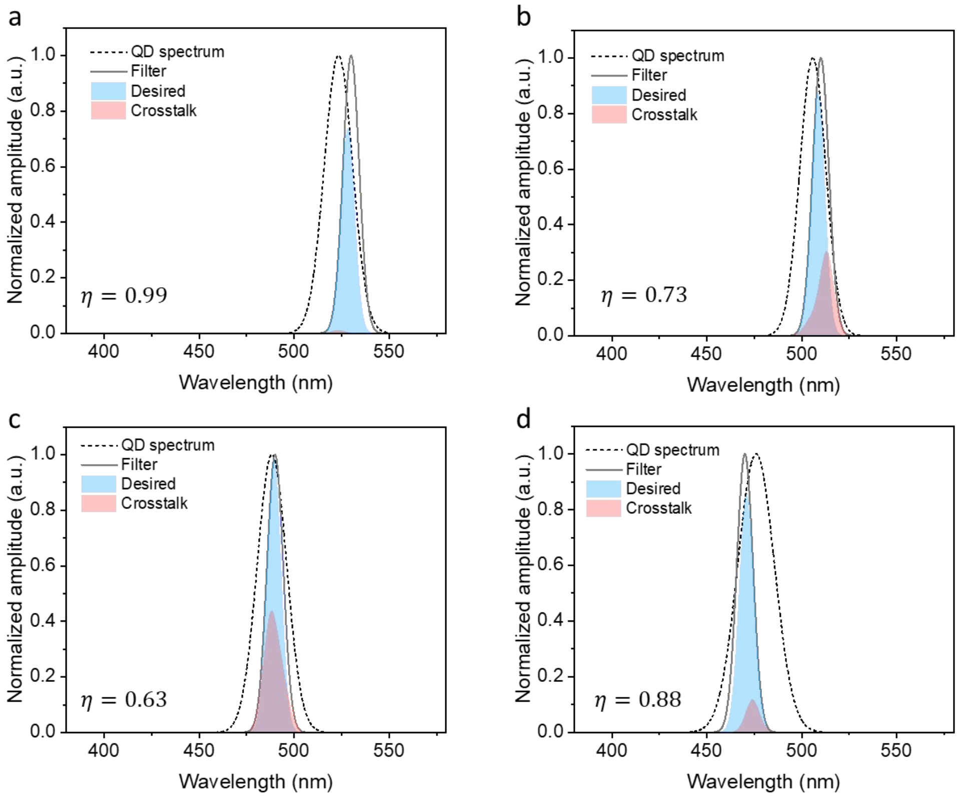

Figure S14. Spectral allocation and crosstalk analysis for QD-based WDM channels for (a) QD1, (b) QD2, (c) QD4, and (d) QD5.
